# A three-dimensional conformal radiofrequency thermocoagulation method for epileptogenic zones in functional cerebral areas: evaluation and prediction of surgical outcomes

**DOI:** 10.1186/s42494-025-00214-6

**Published:** 2025-04-07

**Authors:** Yuchen Gong, Qiangqiang Liu, Yuhai Xie, Puming Zhang, Xiaolai Ye, Wenzhen Chen, Jing Hong, Jiwen Xu, Jun Zhao

**Affiliations:** 1 School of Biomedical Engineering, Shanghai Jiao Tong University, Shanghai, 200240 China; 2 Department of Neurosurgery, Clinical Neuroscience Center Comprehensive Epilepsy Unit, Ruijin Hospital, Shanghai Jiao Tong University School of Medicine, Shanghai, 200025 China; 3 Clinical Neuroscience Center, Ruijin Hospital Luwan Branch, Shanghai Jiao Tong University School of Medicine, Shanghai, 200020 China

**Keywords:** Epilepsy, SEEG, Radiofrequency thermocoagulation, Functional cerebral areas

## Abstract

**Background:**

Stereo-electroencephalography (SEEG)-guided radiofrequency thermocoagulation (RF-TC) emerges as a feasible alternative for drug-resistant focal epilepsy with epileptogenic zones (EZs) located in functional cerebral areas, which is ineligible for resection surgery. However, the efficacy of RF-TC was considered limited. This study described a three-dimensional conformal SEEG-guided RF-TC method to enhance thermocoagulation efficacy and assessed its surgical outcomes in patients with EZs located in functional cerebral areas.

**Methods:**

Ten patients were retrospectively reviewed, and the pathogeneses included focal cortical dysplasia (FCD, *n* = 5) and gray matter heterotopia (GMH, *n* = 5). Three-dimensionally symmetrical electrode placement was designed based on reconstructed cerebral model integrating the etiology and surrounding cerebral cortices, vessels and fibers. The three-dimensional conformal SEEG-guided RF-TC procedure in the functional area was performed by these electrodes penetrating the etiology conformally. Two electrophysiological indicators extracted from SEEG recordings, specifically line length (LL) and approximate entropy (ApEn), were calculated and a support vector machine (SVM) classifier was used to predict outcomes.

**Results:**

Eight patients (80%) achieved Engel class I and International League Against Epilepsy class 1–2 (responders), whereas two patients (20%, one with FCD and one with GMH) did not present long-lasting seizure freedom (non-responders). The sole short-term complication observed was decreased limb muscle strength, with no case of short-term deficits developing into long-term complications. LL and ApEn exhibited different postoperative changes in responders and non-responders. The changes were used to predict outcomes, achieving a 98.05% area under the receiver operating characteristic curve based on SVM.

**Conclusions:**

The three-dimensional conformal SEEG-guided RF-TC method shows a potentially favorable risk–benefit ratio. Postoperative changes of short-term electrophysiological indicators LL and ApEn are promising tools for prognostic prediction.

**Supplementary Information:**

The online version contains supplementary material available at 10.1186/s42494-025-00214-6.

## Background

Antiseizure medication (ASM) is the main treatment for the majority of epilepsy cases. However, individuals with drug-resistant epilepsy are refractory to such therapeutic strategies [1]. For patients with drug-resistant focal epilepsy, surgical resection emerges as a viable option and may lead to clinical improvements or even seizure freedom [2]. Notably, some patients with epileptogenic zones (EZs) located in the functional cortices are ineligible for conventional resection [3]. In response to this clinical challenge, there is an increasing demand for less invasive therapeutic alternatives.

As an alternative method to remove EZs, radiofrequency thermocoagulation (RF-TC) generators harness thermal energy to produce focal necrosis in EZs in a less invasive manner [4]. Stereo-electroencephalography (SEEG), an invasive technique utilizing depth electrodes to record seizure onset and spread directly from the superficial and deeper structures of the brain [5], was first introduced in the 1960s for epilepsy research and later combined with radiofrequency generator in 2004 [3, 6, 7]. Since then, SEEG-guided RF-TC has proven a feasible and safe treatment for epilepsy, especially for patients ineligible for conventional resection surgery [7]. However, outcomes of RF-TC presented less effective might be limited by the spatial sampling of SEEG or the size of coagulation lesions [4, 8]. To improve it, implantation methods have evolved for targeting precisely and coagulating completely [4, 9, 10]. It found that larger thermo-lesion volume in EZs, such as a cross-bonding electrode placement method, could improve thermocoagulation efficacy [9]. In addition to strategic spatial placement of electrodes, augmenting the number of intralesional electrodes has become a preferred method to optimize the lesion volume instead of controlling the RF-TC parameters directly, because it is difficult to monitor real-time parameters in vivo during the RF-TC procedure [3, 11–14]. Increased number of electrodes not only oversamples the presumed tissues with EZs but also enhances the precision of targeting [15]. Further exploration is required for achieving precise and complete ablation of EZs in more complex structures, such as highly functional areas, where the risk–benefit ratio of each electrode must be considered.

A three-dimensional conformal SEEG-guided RF-TC method has been proposed for implanting electrodes in suspicious areas by preoperative noninvasive image assessment and three-dimensional magnetic resonance imaging (MRI) reconstruction, circumscribing the shape of anatomical abnormalities to improve the thermocoagulation efficacy of structural etiology [16]. This method is suitable when the etiology is distributed in a limited area and can be located in imaging assessments. It has been conducted in patients with epilepsy caused by focal cortical dysplasia (FCD) in the eloquent areas, specifically those cases with small volume and confined limits of the etiology, and proven safe and effective compared to unsatisfactory outcomes of traditional RF-TC procedure in FCD. Based on the satisfactory clinical response of the single etiology, in this study, we would take a further investigation for other etiologies. Gray matter heterotopia (GMH) is considered as a kind of more complex structure, for it possibly occurs as contiguous or non-contiguous numerous abnormalities, which brings more difficulties for circumscribing the shape of structural etiology [17, 18]. We hypothesized that the conformal method of SEEG-guided RF-TC could be extended to GMH in functional cerebral areas.

Quantitative analyses of interictal intracranial electroencephalography (EEG) showed the ability to predict long-term seizure outcomes of traditional resection and RF-TC surgery [19–21]. Among existing methods for EEG quantitative analyses, amplitude-frequency and chaotic properties were applicable to describing single-channel epileptic activity patterns of EZs, such as line length (LL) and approximate entropy (ApEn) [22, 23]. We hypothesized the postoperative electrophysiological changes extracted from short-term recordings might serve as clinical predictors of long-term surgical outcomes of the RF-TC procedure. In this study, we aimed to present the surgery improvements of our three-dimensional conformal SEEG-guided RF-TC method and predictors associated with outcomes of this RF-TC method.

## Methods

### Patients

The study included ten patients (four children and six adults) as following criteria: (1) patients suffered from drug-resistant focal epilepsy; (2) the foci of patients involved functional cerebral areas that were poorly accessible to resection surgery with MRI-positive etiologies in the functional cerebral areas, which accorded with SEEG-guided RF-TC indication A (GMH, *n* = 5) and B (FCD, *n* = 5) based on Chinese expert consensus on SEEG-guided RF-TC treatment of drug-resistant epilepsy [24]; (3) patients underwent our three-dimensional conformal RF-TC procedure in Ruijin Hospital, Shanghai Jiao Tong University School of Medicine, from June 2019 to September 2022. In addition to the ten included cases, two patients with hypothalamus hamartoma who underwent the same conformal RF-TC procedure were excluded from the analyses due to the etiology originating outside functional cerebral areas. All patients had taken 2–3 kinds of ASMs before the RF-TC procedure. The baseline clinical details are presented in Table 1. This is a retrospective study that has been approved by the Ruijin Hospital Luwan Branch Ethics Committee (LWEC2023040), Shanghai Jiao Tong University School of Medicine, and has been granted a waiver of informed consent.

**Table 1:** Preoperative clinical features of patients

No.	Sex	Age at RF-TC (year)	Epilepsy duration (year)	Seizure frequency	Seizure types	Side	Epileptogenic focus	MRI etiology	Number of ASMs	Follow-up (month)
1	F	14	3	Daily	FAS/sGTCS	R	Paracentral lobule	FCD	2	64
2	F	25	8	Monthly	FAS	L	Visual cortex	GMH	2	61
3	F	28	12	Weekly	FAS	L	Visual cortex	GMH	2	53
4	F	19	8	Daily	FAS	L	Inferior frontal gyrus	FCD	3	34
5	M	2	1	Daily	FAS	L	Paracentral lobule	FCD	3	26
6	F	24	5	Daily	FIAS	L	Precentral gyrus	FCD	2	49
7	M	25	18	Weekly	FIAS/sGTCS	L	Precentral gyrus	GMH	2	48
8	M	33	25	Daily	FIAS/sGTCS	R	Precentral gyrus	GMH	2	47
9	M	14	12	Monthly	FIAS/sGTCS	R	Precentral gyrus	FCD	2	44
10	M	15	10	Daily	FIAS/sGTCS	L	Inferior parietal lobule	GMH	2	41

*Abbreviation*: *F* female, *M* male, *RF-TC* radiofrequency thermocoagulation, *FAS* focal aware seizure, *sGTCS* secondary generalized tonic–clonic seizure, *FIAS* focal impaired awareness seizure, *R* right, *L* left, *FCD* focal cortical dysplasia, *GMH* gray matter heterotopia, *ASM* antiseizure medication

### Placement of SEEG electrodes

Before implanting the SEEG electrodes, all patients underwent preoperative non-invasive imaging. Pathogenesis was identified using MRI and positron emission tomography (PET) evaluation for further conformal implantation. MRI was performed on a 3.0 T clinical MRI system (Philips Ingenia, Philips Healthcare, Best, Netherlands) using the following acquisition parameters: T1-weighted (TR = 381 ms, TE = 2.3 ms, contiguous 2-mm-thick images), T2-weighted (TR = 3000 ms, TE = 100 ms, contiguous 2-mm-thick images) and FLAIR (TR = 4800 ms, TE = 411.7 ms, contiguous 2-mm-thick images). The details of preoperative MRI images of all cases were shown in Supplementary Fig. 1. No preoperative MRI-negative cases were reported in this study.

**Fig. 1:**
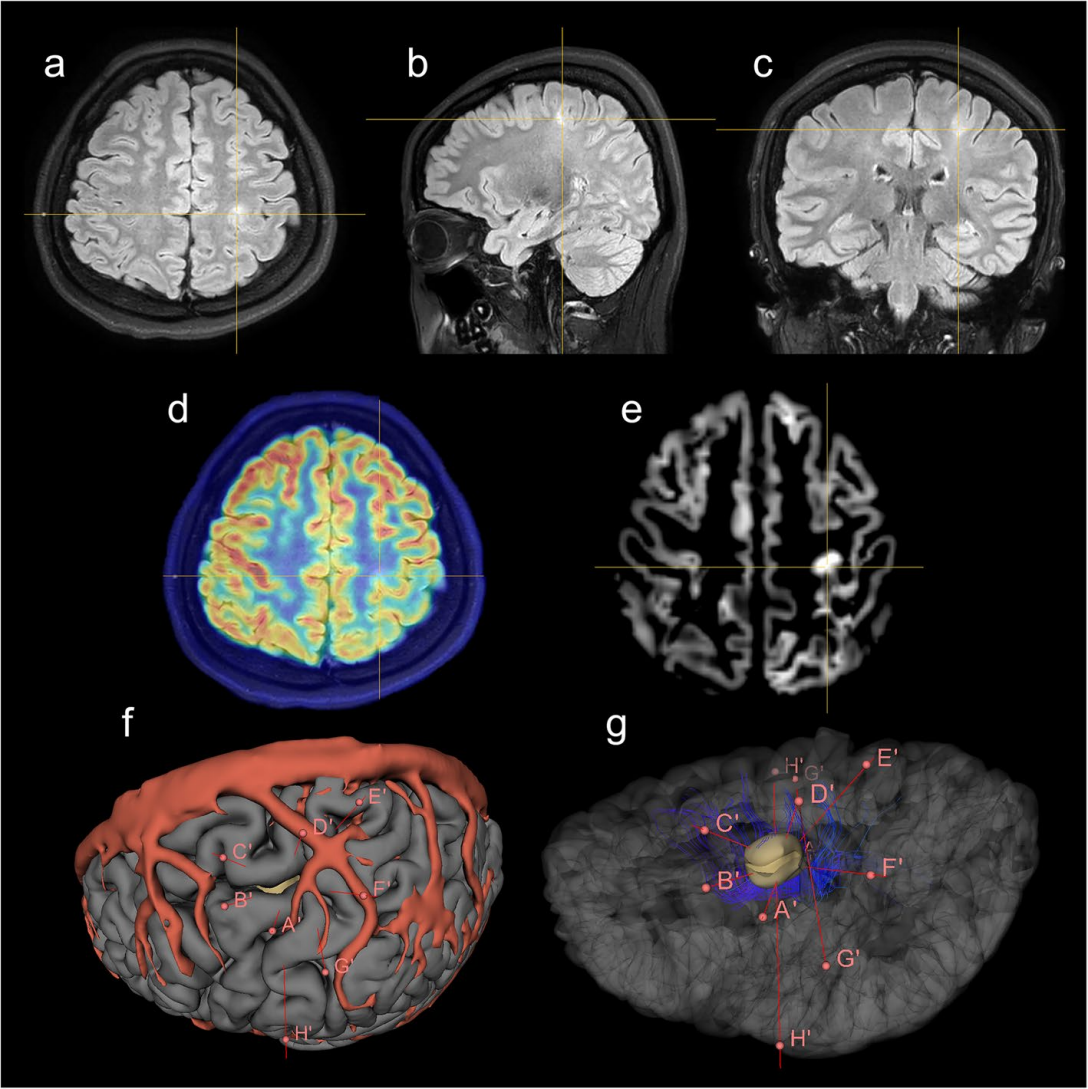
Representative images of electrode implantation designed with image reconstruction (Patient 6). **a**–**c** Preoperative MRI FLAIR images in the axial, sagittal and coronal plane with the FCD in the left precentral gyrus. **d** Fused image of MRI FLAIR and PET with the FCD. **e** Segmentation of gray matters based on MRI T1 images with the FCD. **f**–**g** Reconstruction model of the cerebral surface (gray), vessels (red), electrodes (red lines, with corresponding letters of electrodes), FCD structure (yellow) and corticospinal tract (blue lines), with the personalized SEEG electrode-implantation trajectories based on the models. Crossing point of yellow lines in (**a**–**e**) is the location of the FCD in the left precentral gyrus. Abbreviation: SEEG, stereo-electroencephalography; FCD, focal cortical dysplasia

Conformal implantation of electrodes was designed to completely circumscribe EZs based on the individualized shape of structural abnormalities after noninvasive MRI and PET image investigation (Fig. 1a–e). Surgical modeling of the electrode-implantation was reconstructed on structural MRI, assisted by open-source programs, FreeSurfer (version 7.1) and 3D Slicer (version 4.10.2), to model cerebral hemispheres and lesions, respectively [25, 26]. The details of the MRI structural etiology modeling method were reported in a previous study [27]. Guided by the reconstructed location and shape of the etiology and surrounding cortices, vessels, and fibers, a three-dimensionally symmetrical electrode placement method was designed with intensive electrodes penetrating the etiologic structure conformally from each direction (Fig. 1f–g) for a complete electrophysiological record and effective thermocoagulation. The electrodes were only implanted in the unilateral hemisphere in each case. The number of electrodes was 5–15 without increasing any cost of electrodes compared with the number of electrodes recommended in a previous report which was 7–14 [28, 29].

### SEEG visual assessment

**Fig. 2:**
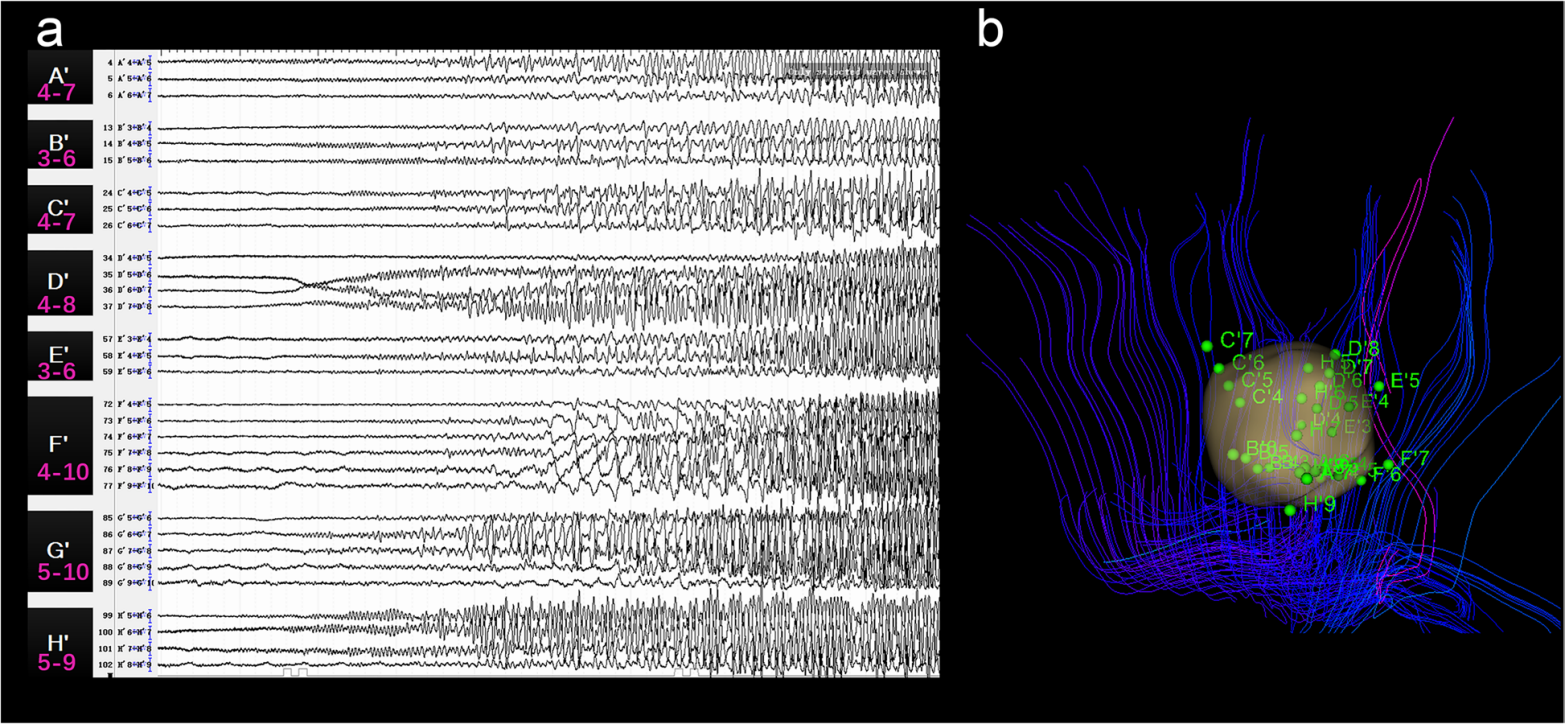
Preoperative SEEG for EZ identification (Patient 6). **a** SEEG signals of identified seizure onset zones in the preictal and ictal period. **b** Spatial relationship between electrode contacts of seizure onset (green dots and corresponding letters of contacts) and the model of FCD (yellow) and corticospinal tracts (blue lines). Abbreviation: SEEG, stereo-electroencephalography; EZ, epileptogenic zones; FCD, focal cortical dysplasia

After electrode implantation following the three-dimensional conformal design, electrical information was recorded by SEEG electrodes (HKHS, Beijing, China). The sampling rates of SEEG ranged from 512 to 2000 Hz. Each electrode was 0.8 mm in diameter with 8–18 recording contacts, 2 mm long and 1.5 mm apart. After three-dimensional implantation of electrodes within the epileptogenic lesion, thermocoagulation was targeted at the EZs identified by two well-established ictal seizure-onset electrophysiological patterns: low-voltage fast activity and low-frequency high-amplitude spiking activity [30]. Cortical electrical stimulation also performed to confirm the function cortices as following parameters: 50 HZ, 3 s, biphasic square-wave current progressively raised from 0.1 to 5.0 mA. Thermocoagulation contacts were determined by ictal pathological discharges and their relationship with the functional cortex and fiber bundle. It was an example of the EZ identification process with the onset and spread patterns in the SEEG ictal recordings of Patient 6 (Fig. 2a), which played a key role in the thermocoagulation decision. Given the high intraoperative risk involving functional cortices and vessels in the functional areas, these areas were circumvented in all cases. All patients underwent intraoperative computed tomography (CT) scanning to verify the position of each electrode and identify any signs of intracranial hemorrhage immediately after implantation.

### Procedure of RF-TC

The RF-TC procedure practice referred to reported guidelines and followed established parameter settings [31]. The distance between contacts was approximately 7 mm for extending thermo-lesions effectively [32]. The RF-TC procedure was performed using an R-2000B radiofrequency lesion generator (BNS, Beijing, China). Thermo-lesions were produced between bipolar contacts identified as thermocoagulation contacts by a 3.5 W current power, which created lesions with 5–7 mm in diameter, and enlarged by contacts of two adjacent electrodes. This process lasted for 60 s, and the SEEG was monitored after the procedure. If the persistence of seizure or continued presence of the primary interictal discharges prior to the ablation existed, another session of RF-TC procedure would be performed. None of the patients was under anesthesia during the process.

### Electrophysiological and statistical analysis

Two electrophysiological features, LL and ApEn, were calculated to identify electrophysiological properties before and after the RF-TC procedure. LL describes the changes in signal amplitude and frequency, and ApEn measures the probability of generating a new pattern in a time series [33]. SEEG signals with obvious contamination were rejected manually before preprocessing. Channels of RF-TC contacts were removed from postoperative data because only noise remained in those contacts after the thermocoagulation of brain tissues. The average reference montage was used for the analysis. SEEG recordings were filtered into the filtered band (0.5–250 Hz) and three sub-bands including the low-frequency band (0.5–50 Hz), high-gamma band (50–80 Hz) and ripple band (80–250 Hz). Notch filters removed the 50 Hz power interference and odd harmonics in the signals. Signals at a 1000 or 2000 Hz sampling rate were resampled to 500 Hz. Features were extracted from 200 s interictal SEEG signals with a 10 s non-overlapping window before and after the RF-TC procedure. Before statistical analysis, the features were normalized using within-subject z-scoring to adjust for inter-individual differences in mean and standard deviation [34].

To evaluate the differences between preoperative features of signals from RF-TC contacts and those of others, Mann–Whitney U test was performed because of the non-Gaussian distribution. To compare the changes between the preoperative features and the postoperative, Wilcoxon signed-rank test was performed with the average features of each contact. Bonferroni’s correction was used for multiple comparisons. The results of the one-tailed test (*P* < 0.05 after correction) were considered statistically significant when the preoperative electrophysiological feature values were higher than the postoperative (right-tailed) or less than the postoperative (left-tailed), and when the preoperative electrophysiological feature values of signals from RF-TC contacts were higher than those of other contacts. The calculation was performed with Matlab R2022a (MathWorks Inc., Natick, MA, USA).

### Prediction model construction and assessment

The ability for surgical outcome prediction of the electrophysiological biomarkers was investigated by support vector machine (SVM). The sensor-level features to train the prediction model were defined as the preoperative biomarkers from each contact minus the corresponding postoperative biomarkers, including LL and ApEn from four frequency bands, as eight features available in total. The sensor-level data had two labels for classification: the positive class from responders (patients with Engel class I) and the negative class from non-responders (patients with other Engel classes). The individual-level classification predictor, the response possibility, was defined as the intra-individual percentage of positive contacts.

At the sensor level, the dataset included 834 positive samples from eight responders and 163 negative samples from two non-responders. It was randomly divided into the training set (695 positive and 136 negative) and the test set (139 positive and 27 negative). The positive samples were subdivided into five subsets in the training set to fix the unbalanced dataset. The best training subset and hyperparameters were determined by a 25-fold cross-validation of each responder’s training subset and the non-responders’ training set based on the highest average of the area under the receiver operating characteristic curve (AUC). The best classifier was assessed by the test set with comprehensive measures (accuracy, precision, recall, F1-score, AUC) [35]. The prediction SVM model was performed with Python (version 3.7.6) with scikit-learn (version 0.22.1) package [36].

## Results

### Outcome after RF-TC procedure

**Fig. 3:**
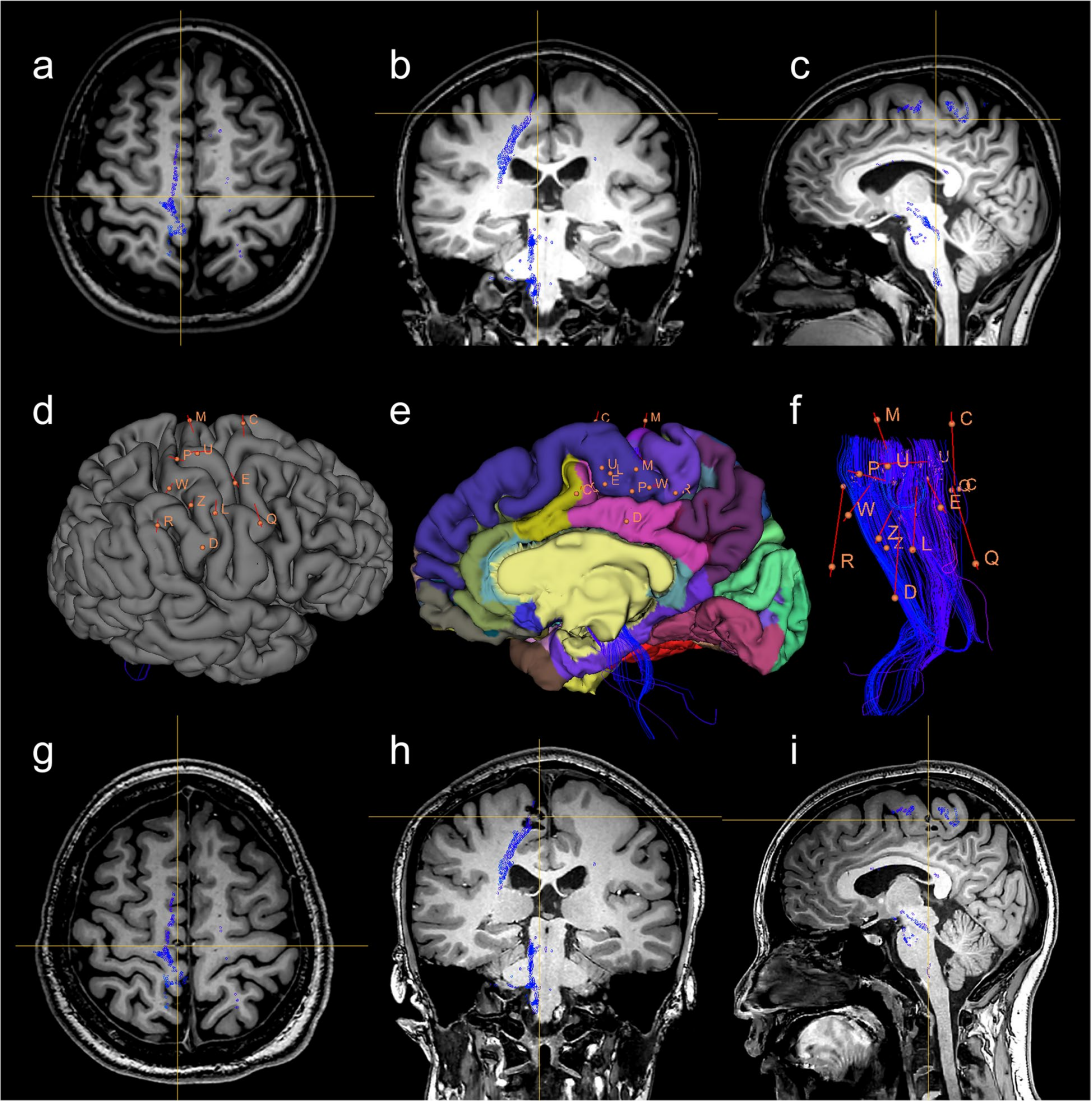
Preoperative and postoperative image evaluation (Patient 1). **a**–**c** Preoperative MRI T1 images showing the location of thermo-lesions in the right paracentral lobule in the axial, coronal and sagittal plane fused with the corticospinal tract of the lower limb. **d** Lateral view of spatial relationship between electrodes and the three-dimensional cerebral model. **e** Medial view of spatial relationship between the targets of electrodes and the three-dimensional cerebral model, with cortical parcellation marked by different colors. **f** Spatial relationship between trajectories of electrodes and right corticospinal tract of the lower limb. **g**–**i** Postoperative MRI T1 showing the range of thermo-lesions in the axial, coronal and sagittal plane fused with the right corticospinal tract of the lower limb. Blue streamlines in (**a**–**c**) and (**g**–**i**), and blue lines in (**d**–**f**) are the right corticospinal tract of the lower limb. Crossing point of yellow lines in (**a**–**c**) and (**g**–**i**) is the location of thermo-lesions. Orange lines in (**d**–**f**) present electrodes with their corresponding letters

In our study, all patients underwent electrode implantation and two sessions of thermocoagulation in the functional cerebral areas. The details of electrode implantation and surgical outcomes after the RF-TC procedure are listed in Table 2. The location of the thermocoagulation contacts covered the precentral gyrus (*n* = 4) and paracentral lobule (*n* = 2), visual cortex (*n* = 2), inferior parietal lobule (*n* = 1), and inferior frontal gyrus (*n* = 1). Pathogeneses revealed by preoperative MRI included FCD (*n* = 5) and GMH (*n* = 5). The number of RF-TC contacts in each patient ranged from 7 to 76. An example was shown in Fig. 3, including the preoperative investigation and electrode implantation (Fig. 3a–f) and the range of thermo-lesions after complete thermocoagulation (Fig. 3g–i) from Patient 1 with GMH.

**Table 2:** SEEG-guided RF-TC surgical data and outcomes of patients

No.	Sampling rate (Hz)	Number of electrodes	Number of RF-TC electrodes	Number of RF-TC contacts	RF-TC contacts	Short-term complications	Engel class	ILAE class
1	1000	11	3	14	M 1–4, P 1–5, W 1–5	Decreased left lower limb muscle strength	I	1
2	2000	6	6	26	A 3–5, B 4–8, C 4–10, D 5–8, E 7–10, F 1–3	N/A	I	1
3	512	5	5	39	A' 2–4, B' 5–8, C' 4–17, D' 1–15, E' 1–3	N/A	III	5
4	2000	13	1	7	C' 4–10	N/A	I	1
5	2000	11	3	16	C' 1–5, B' 4–12, K' 1–2	N/A	I	1
6	1000	8	7	26	A' 4–7, B' 4–7, C' 3–6, D' 5–8, E' 3–5, F' 5–6, H' 5–9	Decreased right upper limb muscle strength	I	1
7	2000	12	5	39	A' 4–7, B' 1–5, C' 1–10, F' 1–12, L' 4–11	Decreased right upper limb muscle strength	I	2
8	1000	13	10	62	A 1–5, B 1–12, C 1–10, D 1–5, E 1–5, F 1–6, G 1–6, J 1–5, K 1–5, L 1–3	Decreased left upper limb muscle strength	I	1
9	1000	11	5	36	C 3–9, E 6–13, K 1–6, L 7–13, P 7–14	Decreased left upper limb muscle strength	II	4
10	2000	15	10	76	D’ 1–10, E’ 1–15, F’ 1–12, G’ 4–11, H’ 7–11, I’ 7–11, L’ 5–9, M’ 3–7, N’ 2–6, P’ 3–8	N/A	I	1

*Abbreviation*: *SEEG* stereo-electroencephalography, *RF-TC* radiofrequency thermocoagulation, *ILAE* International League Against Epilepsy, *N/A* not applicable

The follow-up period ranged from 26 to 64 months. None of the patients underwent conventional resection surgery or stopped taking ASM after the procedure. Outcomes were assessed by Engel and International League Against Epilepsy (ILAE) outcome scale [37] and eight patients (80%) achieved Engel class I and ILAE class 1–2. Two patients (20%) did not show significant improvement in the most recent follow-up after the procedure, including one case with GMH (Patient 3) and one case with FCD (Patient 9). Because the right GMH did not lead to any seizure, the electrode implantation and thermocoagulation in Patient 3 with bilateral GMH was planned only in the left hemisphere. The unsatisfactory outcome might be attributed to this extensive size of the GMH, which made it impossible for the SEEG electrodes to fully cover the affected area, resulting in incomplete coagulation of the structural abnormality. Patient 9 with FCD was seizure-free for a while post-procedure but experienced recurrence during the 15-month follow-up period.

### Safety

None of the patients presented with intracranial hemorrhage or infection. Half of the patients showed short-term postoperative neurological deficits, including decreased contralateral upper (*n* = 4) or lower (*n* = 1) limb muscle strength (Table 2). All of the cases with short-term deficits in the upper limbs were coagulated in the precentral gyrus, and the case with short-term deficits in the lower limb was coagulated in the paracentral lobule. Both regions were located in the central lobe associated with motor function. Therefore, the short-term deficits in this study were foreseeable. In four patients, these deficits were completely resolved and did not develop long-term functional decline. One patient (Patient 7) suffered a longer-term motor impairment in the distal parts of the right hand, and finally recovered within 16 months. None of cases with neurological deficits developed permanent functional impairments. Two RF-TC surgical factors of patients with short-term functional deficits (*n* = 5), the number of electrodes and contacts with RF-TC, were compared with those of patients without functional deficits (*n* = 5) by Mann–Whitney U test. The number of electrodes and RF-TC contacts did not show significant difference between patients with and without deficits (*P* = 0.89 and 0.81 respectively, two-tailed, Supplementary Fig. 2).

### Electrophysiological analysis

**Fig. 4:**
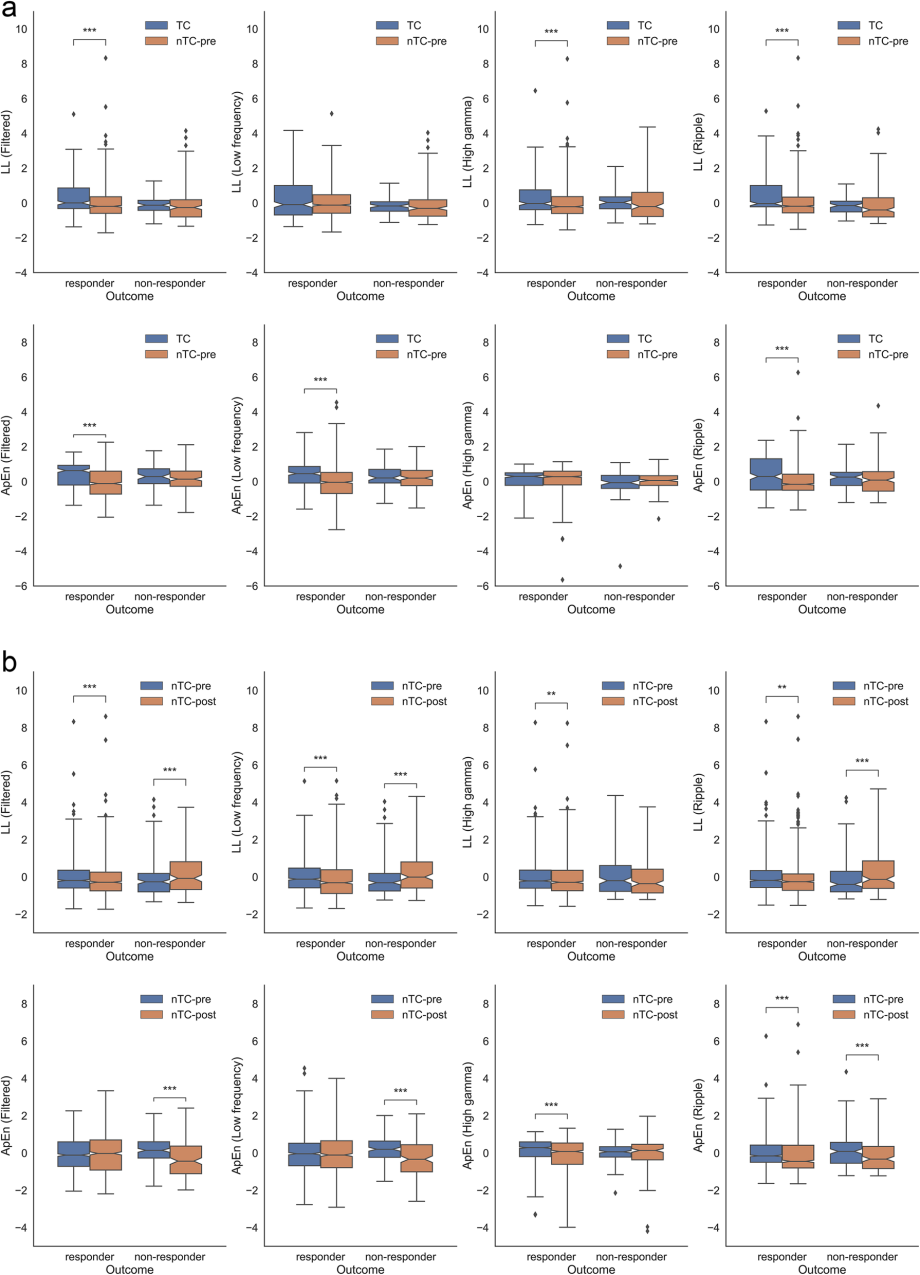
Statistical analysis of LL and ApEn. **a** LL and ApEn between the RF-TC contacts and the other contacts in the preoperative interictal period. **b** LL and ApEn between preoperative and postoperative contacts without RF-TC. For all notched boxplots, the central mark of the box indicates the median, and the bottom and top edges of the box indicate the 25th and 75th percentiles, respectively. The notches represent the confidence interval around the median. The lower and upper whiskers describe the rest of the distribution. Individual points beyond the whiskers of the boxplot are marked as outliers. Asterisks represent two-tailed significant p-values with Bonferroni’s correction (*, 0.01 ≤ *P* < 0.05; **, 0.001 ≤ *P* < 0.01; ***, *P* < 0.001). Abbreviation: LL, line length; ApEn, approximate entropy; RF-TC, radiofrequency thermocoagulation; TC, biomarkers from RF-TC contacts; nTC-pre, preoperative biomarkers from contacts without RF-TC; nTC-post, postoperative biomarkers from contacts without RF-TC

The statistical results between contacts with and without RF-TC are shown in Fig. 4a. LL of responders in the filtered band, high-gamma and ripple band was significantly higher in RF-TC contacts than in the other contacts in the preoperative interictal period. ApEn of responders in the filtered, low-frequency and ripple band was significantly higher in RF-TC contacts than in the other contacts. No significant difference between the biomarkers in RF-TC contacts and the other contacts was found in non-responders. The significant differences between contacts with and without RF-TC in responders suggested the underlying aberrant electrophysiology in the structure conformally coagulated in this study.

As shown in Fig. 4b, in responders, LL in all four bands and ApEn in the high-gamma and ripple band significantly decreased in the postoperative interictal signal compared with the preoperative. In contrast, in non-responders, LL in the high-gamma band did not show any significant difference and other bands showed significantly higher LL in the postoperative period. ApEn in non-responders in the filtered and low-frequency band showed a significant decline in the postoperative period when the biomarkers did not show any significant changes in responders. Only ApEn in the ripple band showed the same significant postoperative decline in two groups with different surgical outcomes. The different postoperative changes between the responders and non-responders suggested the potential of these two electrophysiological biomarkers for outcome prediction.

### Prediction model evaluation

The SVM model was trained with the LL and ApEn features for the prediction task. The evaluation measures of the classifier during cross-validation by training subsets are listed in Table 3, with good sensor-level predictive performance of 98.1% AUC in the test set by the best classifier. The individual-level outcomes were predicted by the best sensor-level classification. In the individual-level prediction, the patient was predicted as a responder when the response possibility was over 50%, otherwise, the patient was classified as a non-responder. The best classifier showed an accurate outcome prediction performance for all the responders and non-responders, as shown in Table 3. All responders and non-responders were classified accurately in the individual-level prediction, with 61.5–100% and 5–14.3% response possibility respectively. This result suggested LL and ApEn features had potential not only for the sensor-level prediction but for individual-level prediction.

**Table 3:** Cross-validation performance and evaluation of the prediction model with best hyperparameters and training subsets

Sensor-level (training set)				
Accuracy	Precision	Recall	F1-score	AUC
0.934	0.934	0.934	0.934	0.985
Sensor-level (test set)				
Accuracy	Precision	Recall	F1-score	AUC
0.886	0.886	0.886	0.895	0.981
Individual-level				
Patient number	Outcome	Response possibility		Predicted outcome
1	responder	0.963		responder
2	responder	0.857		responder
3	non-responder	0.143		non-responder
4	responder	0.96		responder
5	responder	0.696		responder
6	responder	1		responder
7	responder	0.615		responder
8	responder	1		responder
9	non-responder	0.05		non-responder
10	responder	0.875		responder

*Abbreviation*: *AUC* the area under the receiver operating characteristic curve

## Discussion

FCD and GMH are two kinds of malformation of cortical development, which are major etiologies of drug-resistant epilepsy [38]. In previous traditional SEEG-guided RF-TC studies, patients with FCD experienced a seizure freedom rate of 18–52% [39, 40], and 46–76% of patients with GMH showed seizure freedom with Engel class I or ILAE class 1 [41–43], and by using the conformal RF-TC method, outcomes of FCD cases were improved effectively that 77.3% patients achieved seizure-free [16]. In this study, a three-dimensional conformal SEEG-guided RF-TC was performed for ten patients with different structural etiologies involving functional cortex and inaccessible to resection surgery, including FCD (*n* = 5) and GMH (*n* = 5). To facilitate conformal thermocoagulation in these etiologies, a three-dimensional symmetrical electrode placement method was designed, which provides possibilities for enhancing coagulation efficacy and mitigating the risk of secondary procedures in functional cortical EZs. In this study, 80% of patients became Engel class I and ILAE class 1–2 after the procedure, including 75% of children (*n* = 3) and 83% of adults (*n* = 5), 80% of patients with FCD (*n* = 4) and 80% of patients with GMH (*n* = 4), which showed good clinical efficacy in patients of different ages and with different etiologies. Compared with previous studies, both FCD and GMH cases showed a higher percentage of Engel class I in this study, which suggested the three-dimensional conformal SEEG-guided RF-TC method could improve surgical outcomes in both etiologies. However, due to the limited sample size in this study, its advantages over reported RF-TC methods need further validation with more surgical cases.

Minimization of irreversible neurological deficits is an advantage of SEEG-guided RF-TC over traditional open surgery [44]. However, side effects like edema, hemorrhage and functional deficits may develop especially in a limited brain volume with concentrated lesions and electrodes [11, 45]. In our study, conformal RF-TC did not result in any intracranial hemorrhage or infection. Surgery in five cases in functional areas led to short-term decreased limb muscle strength associated with the precentral gyrus (*n* = 4) and paracentral lobule (*n* = 1), which resolved with progressive edema clearance and did not develop into long-term complications. The location of electrodes was considered as the eloquent areas in all ten cases, including the paracentral lobule, visual cortex, inferior frontal gyrus, precentral gyrus and inferior parietal lobule. However, except the short-term motor deficits, we did not find other impaired function related to the eloquent cortex, such as language, memory and cognition and vision. Thus, the method showed satisfactory outcomes to avoid impairment in the eloquent cortex. Furthermore, we did not find significant difference in the number of electrodes and RF-TC contacts between patients with (*n* = 5) and without short-term deficits (*n* = 5). In this study, because electrodes were concentrated in the etiologies within the eloquent areas, the number of electrodes and RF-TC contacts could indicate the local density of electrodes and ablation in the eloquent areas. The difference without statistical significance suggested that although risks of locally increased number of electrodes and overzealous ablation should be considered before the procedure, their exact association with the operative side-effects on the eloquent cortex still needed further evidence when more samples are added in the future. Evaluating outcomes from perspectives on efficacy and safety, we proved that the three-dimensional conformal SEEG-guided RF-TC might be a feasible therapeutic strategy for patients with EZs located in the functional areas with a commendable risk–benefit ratio.

The most conventional frequency band for routine clinical research is the low-frequency band (0.5–50 Hz) [46]. With the application of intracranial EEG such as SEEG, higher spatial and temporal resolutions give insights into epileptic activities in higher frequency bands (> 50 Hz) and provide biomarkers like high-frequency oscillations (ripples: 80–250 Hz, fast ripples: 250–500 Hz) for EZ localization and resection outcome prediction [47–49]. We did not analyze signals in the fast-ripple band (250–500 Hz) in this study because of the low sampling rate (512 Hz) in one non-responder case (Patient 3). Further exploration of electrophysiological biomarkers in the fast ripple band is expected, as fast ripples exhibit greater specificity for pathological activities compared to ripples, which may also occur frequently in physiological activities in some functional areas [50]. It is feasible when all SEEG signals are sampled at a high sampling rate (≥ 1000 Hz) in future cases.

The previous SEEG study found that the property of electrode contacts inside nodular heterotopia was different from those of contacts outside [51]. In the analysis of preoperative signals, we found LL (in the filtered, high-gamma and ripple band) and ApEn (in the filtered, low frequency and ripple band) in RF-TC contacts were significantly higher than others in responders. The significant differences we found between contacts with and without thermocoagulation were in line with the implementation of LL and ApEn into automatic seizure detection and EZ localization [33, 52, 53]. Higher LL suggested higher activity and higher ApEn reflected more irregular patterns occurring in the SEEG signals [54, 55]. The dynamical state of interictal epileptiform discharge, a classic biomarker of epileptic brain tissues caused by synchronous neural activities, was thought to play multifaceted roles in seizure genesis [56]. The electrophysiological patterns with higher and irregular interictal activity in RF-TC contacts as EZs, compared with other brain tissues, might be related to similar physiological mechanisms with seizure onset in our study, that suggested they were more fragile for epileptogenesis. Compared with successful conformal thermocoagulation of etiologies in responders, no significant difference between RF-TC contacts and the others was found in two non-responders. It might suggest that some EZs remained outside the thermocoagulation-targeted etiology in non-responders, leading to their unsatisfactory long-term outcomes. The case of Patient 3, a non-responder who suffered from bilateral GMH but underwent implantation and thermocoagulation in the left hemisphere, might support this explanation to some extent.

Based on different statistical results between the postoperative signal changes of contacts without RF-TC in responders and non-responders, we took the prediction task with the changes between preoperative and short-term postoperative LL and ApEn in four frequency bands and achieved 100% accuracy at the individual level, affirming the predictive ability of preoperative and short-term postoperative LL and ApEn for thermocoagulation outcomes. The usefulness of LL and ApEn for outcome prediction will inspire studies of the prediction abilities of other electrophysiological fractal and chaotic features, which have been applied in other epilepsy-related classification tasks [53]. Furthermore, it has the potential to predict RF-TC surgical outcomes in a comprehensive aspect by integrating electrophysiological indicators and traditional clinical characteristics related to good outcomes [57, 58]. In this study, we chose SVM for training the prediction model. To train the SVM classifier is to find the optimal hyperplane between two classes, which is relatively mathematically interpretable and suitable for moderate data size with relatively less computational complexity [59, 60]. The satisfying prediction outcome indicated the computational ability of SVM with small samples and features. Cross-validation used in the study could avoid the model overfitting the data because the test set was unseen during training the model [61]. However, for the present limited sample size, more cases are needed to further validate the generalization of the prediction model. Other learning models like neural network can be used in the future for conditions with more complexity when more features are added into the analyses and the sample size is large enough [62].

There are limitations to our study because of the limited number of cases. We only conducted the surgery in cases with FCD and GMH, and did not apply this method to more epileptogenic etiologies like polymicrogyria. The study of the association between clinical factors and surgical outcomes was not available unless more individual-level cases were included. The generalization of the prediction model still needed validating to avoid potential overfitting problem. Further studies are still needed for the physiological interpretation of the prediction abilities of features.

## Conclusions

Our results suggest a promising risk–benefit ratio for the three-dimensional conformal SEEG-guided RF-TC method in the treatment of epilepsy with EZ located in the functional areas. In this study, postoperative changes of LL and ApEn appeared to serve as valuable predictors for long-term postoperative outcomes.

## Supplementary Information

Supplementary Material 1.

## Data Availability

Data sharing is not applicable to this article as no datasets were generated.

## Abbreviations

ApEn: Approximate entropy
ASM: Antiseizure medication
AUC: Area under the receiver operating characteristic curve
CT: Computed tomography
EEG: Electroencephalography
EZs: Epileptogenic zones
FCD: Focal cortical dysplasia
GMH: Gray matter heterotopia
ILAE: International League Against Epilepsy
LL: Line length
MRI: Magnetic resonance imaging
PET: Positron emission tomography
RF-TC: Radiofrequency thermocoagulation
SEEG: Stereo-electroencephalography
SVM: Support vector machine

## References

[1] Thijs RD, Surges R, O’Brien TJ, Sander JW. Epilepsy in adults. Lancet. 2019;393(10172):689–701. 30686584 10.1016/S0140-6736(18)32596-0

[2] Spencer S, Huh L. Outcomes of epilepsy surgery in adults and children. Lancet Neurol. 2008;7(6):525–37. 18485316 10.1016/S1474-4422(08)70109-1

[3] Bourdillon P, Isnard J, Catenoix H, Montavont A, Rheims S, Ryvlin P, et al. Stereo-electro-encephalography-guided radiofrequency thermocoagulation: from in vitro and in vivo data to technical Guidelines. World Neurosurg. 2016;94:73–9. 27373418 10.1016/j.wneu.2016.06.095

[4] Englot DJ, Birk H, Chang E. Seizure outcomes in nonresective epilepsy surgery: an update. Neurosurg Rev. 2017;40(2):181–94. 27206422 10.1007/s10143-016-0725-8 PMC5118196

[5] Zijlmans M, Zweiphenning W, van Klink N. Changing concepts in presurgical assessment for epilepsy surgery. Nat Rev Neurol. 2019;15(10):594–606. 31341275 10.1038/s41582-019-0224-y

[6] Bancaud J, Talairach J. Methodology of stereo EEG exploration and surgical intervention in epilepsy. Rev Otoneuroophtalmol. 1973;45(4):315–28. 4603118

[7] Guenot M, Isnard T, Ryvlin T, Fischer T, Mauguiere T, Sindou M. SEEG-guided RF thermocoagulation of epileptic foci: feasibility, safety, and preliminary results. Epilepsia. 2004;45(11):1368–74. 15509237 10.1111/j.0013-9580.2004.17704.x

[8] Kakisaka Y, Kubota Y, Wang ZI, Piao Z, Mosher JC, Gonzalez-Martinez J, et al. Use of simultaneous depth and MEG recording may provide complementary information regarding the epileptogenic region. Epileptic Disord. 2012;14(3):298–303. 22940092 10.1684/epd.2012.0517

[9] Fan XT, Shan YZ, Lu C, An Y, Wang YH, Du JL, et al. Optimized SEEG-guided radio frequency thermocoagulation for mesial temporal lobe epilepsy with hippocampal sclerosis. Seizure. 2019;71:304–11. 31521052 10.1016/j.seizure.2019.08.011

[10] Bourdillon P, Rheims S, Catenoix H, Montavont A, Ostrowsky-Coste K, Isnard J, et al. Surgical techniques: Stereoelectroencephalography-guided radiofrequency-thermocoagulation (SEEG-guided RF-TC). Seizure. 2020;77:64–8. 30711397 10.1016/j.seizure.2019.01.021

[11] Cossu M, Cardinale F, Casaceli G, Castana L, Consales A, D’Orio P, et al. Stereo-EEG-guided radiofrequency thermocoagulations. Epilepsia. 2017;58(Suppl. 1):66–72. 28386919 10.1111/epi.13687

[12] Bourdillon P, Isnard J, Catenoix H, Montavont A, Rheims S, Ryvlin P, et al. Stereo electroencephalography-guided radiofrequency thermocoagulation (SEEG-guided RF-TC) in drug-resistant focal epilepsy: Results from a 10-year experience. Epilepsia. 2017;58(1):85–93. 27859033 10.1111/epi.13616

[13] Mullatti N, Landre E, Mellerio C, Oliveira AJ, Laurent A, Turak B, et al. Stereotactic thermocoagulation for insular epilepsy: lessons from successes and failures. Epilepsia. 2019;60(8):1565–79. 31206643 10.1111/epi.16092

[14] Catenoix H, Mauguiere F, Guenot M, Ryvlin P, Bissery A, Sindou M, et al. SEEG-guided thermocoagulations: a palliative treatment of nonoperable partial epilepsies. Neurology. 2008;71(21):1719–26. 19015488 10.1212/01.wnl.0000335166.20451.88

[15] Bourdillon P, Devaux B, Job-Chapron AS, Isnard J. SEEG-guided radiofrequency thermocoagulation. Neurophysiol Clin. 2018;48(1):59–64. 29273383 10.1016/j.neucli.2017.11.011

[16] Guo Q, Tan HP, Chen J, Chen MB, Zhang LM, Zhang W, et al. Efficacy and safety of conformal thermocoagulation guided by stereotactic electroencephalogram in the treatment of epilepsy caused by focal cortical dysplasia in eloquent cortex. Zhonghua Yi Xue Za Zhi. 2021;101(41):3393–8. (in Chinese). 10.3760/cma.j.cn112137-20210418-00927 34758542

[17] Guerrini R, Dobyns WB. Malformations of cortical development: clinical features and genetic causes. Lancet Neurol. 2014;13(7):710–26. 24932993 10.1016/S1474-4422(14)70040-7 PMC5548104

[18] Cossu M, Mirandola L, Tassi L. RF-ablation in periventricular heterotopia-related epilepsy. Epilepsy Res. 2018;142:121–5. 28705474 10.1016/j.eplepsyres.2017.07.001

[19] Klimes P, Peter-Derex L, Hall J, Dubeau F, Frauscher B. Spatio-temporal spike dynamics predict surgical outcome in adult focal epilepsy. Clin Neurophysiol. 2022;134:88–99. 34991017 10.1016/j.clinph.2021.10.023

[20] Asano E, Juhasz C, Shah A, Sood S, Chugani HT. Role of subdural electrocorticography in prediction of long-term seizure outcome in epilepsy surgery. Brain. 2009;132:1038–47. 19286694 10.1093/brain/awp025 PMC2668945

[21] Simula S, Garnier E, Contento M, Pizzo F, Makhalova J, Lagarde S, et al. Changes in local and network brain activity after stereotactic thermocoagulation in patients with drug-resistant epilepsy. Epilepsia. 2023;64(6):1582–93. 37032394 10.1111/epi.17613

[22] Pizarro D, Ilyas A, Chaitanya G, Toth E, Irannejad A, Romeo A, et al. Spectral organization of focal seizures within the thalamotemporal network. Ann Clin Transl Neur. 2019;6(9):1836–48. 10.1002/acn3.50880 PMC6764631 31468745

[23] Marin AL, Taralunga DD, Neagu GM. Signal processing techniques for the localization of seizure-onset zones in the brain. IEEE Int Sym Med Mea. 2024. 10.1109/MeMeA60663.2024.10596857.

[24] Functional Neurosurgery Group of the Neurosurgery Branch of the Chinese Medical Association, China Association Against Epilepsy, Committee of Experts in Neurosurgical Robot Application Demonstration Project of China. Chinese Expert Consensus on Stereo-electroencephalography-guided Radiofrequency Thermocoagulation Treatment of Drug-resistant Epilepsy. Zhonghua Yi Xue Za Zhi. 2021;101(29):2276–82. (in Chinese).

[25] Fischl B. FreeSurfer. Neuroimage. 2012;62(2):774–81. 22248573 10.1016/j.neuroimage.2012.01.021 PMC3685476

[26] Fedorov A, Beichel R, Kalpathy-Cramer J, Finet J, Fillion-Robin JC, Pujol S, et al. 3D Slicer as an image computing platform for the quantitative imaging network. Magn Reson Imaging. 2012;30(9):1323–41. 22770690 10.1016/j.mri.2012.05.001 PMC3466397

[27] Liu Q, Wang J, Wang C, Wei F, Zhang C, Wei H, et al. FreeSurfer and 3D slicer-assisted SEEG implantation for drug-resistant epilepsy. Front Neurorobot. 2022;16:848746. 35295674 10.3389/fnbot.2022.848746 PMC8918516

[28] Mullin JP, Shriver M, Alomar S, Najm I, Bulacio J, Chauvel P, et al. Is SEEG safe? A systematic review and meta-analysis of stereo-electroencephalography-related complications. Epilepsia. 2016;57(3):386–401. 26899389 10.1111/epi.13298

[29] Chassoux F, Navarro V, Catenoix H, Valton L, Vignal JP. Planning and management of SEEG. Neurophysiol Clin. 2018;48(1):25–37. 29254835 10.1016/j.neucli.2017.11.007

[30] Perucca P, Dubeau F, Gotman J. Intracranial electroencephalographic seizure-onset patterns: effect of underlying pathology. Brain. 2014;137(1):183–96. 24176980 10.1093/brain/awt299

[31] Isnard J, Taussig D, Bartolomei F, Bourdillon P, Catenoix H, Chassoux F, et al. French guidelines on stereoelectroencephalography (SEEG). Neurophysiol Clin. 2018;48(1):5–13. 29277357 10.1016/j.neucli.2017.11.005

[32] Wang D, Wei P, Shan Y, Ren L, Wang Y, Zhao G. Optimized stereoelectroencephalography-guided radiofrequency thermocoagulation in the treatment of patients with focal epilepsy. Ann Transl Med. 2020;8(1):15. 32055606 10.21037/atm.2019.10.112 PMC6995730

[33] An N, Ye XL, Liu QQ, Xu JW, Zhang PM. Localization of the epileptogenic zone based on ictal stereo-electroencephalogram: Brain network and single-channel signal feature analysis. Epilepsy Res. 2020;167:106475. 33045665 10.1016/j.eplepsyres.2020.106475

[34] Wagstyl K, Adler S, Pimpel B, Chari A, Seunarine K, Lorio S, et al. Planning stereoelectroencephalography using automated lesion detection: Retrospective feasibility study. Epilepsia. 2020;61(7):1406–16. 32533794 10.1111/epi.16574 PMC8432161

[35] Flach PA, Kull M. Precision-recall-gain curves: PR analysis done right. Adv Neural Inf Process Syst. 2015;28:838–46.

[36] Pedregosa F, Varoquaux G, Gramfort A, Michel V, Thirion B, Grisel O, et al. Scikit-learn: machine learning in python. J Mach Learn Res. 2011;12:2825–30.

[37] Wieser HG, Blume WT, Fish D, Goldensohn E, Hufnagel A, King D, et al. ILAE Commission Report. Proposal for a new classification of outcome with respect to epileptic seizures following epilepsy surgery. Epilepsia. 2001;42(2):282–6. 11240604

[38] Severino M, Geraldo AF, Utz N, Tortora D, Pogledic I, Klonowski W, et al. Definitions and classification of malformations of cortical development: Practical guidelines. Brain. 2020;143(10):2874–94. 32779696 10.1093/brain/awaa174 PMC7586092

[39] Bourdillon P, Cucherat M, Isnard J, Ostrowsky-Coste K, Catenoix H, Guénot M, et al. Stereo-electroencephalography-guided radiofrequency thermocoagulation in patients with focal epilepsy: a systematic review and meta-analysis. Epilepsia. 2018;59(12):2296–304. 30345535 10.1111/epi.14584

[40] Li YM, Gao JY, Ye Z, Mu J. Magnetic resonance-guided laser interstitial thermal therapy vs. stereoelectroencephalography-guided radiofrequency thermocoagulation in epilepsy patients with focal cortical dysplasia: a systematic review and meta-analysis. Front Neurol. 2023;14:1241763. 37928136 10.3389/fneur.2023.1241763 PMC10625445

[41] Mirandola L, Mai RF, Francione S, Pelliccia V, Gozzo F, Sartori I, et al. Stereo-EEG: diagnostic and therapeutic tool for periventricular nodular heterotopia epilepsies. Epilepsia. 2017;58(11):1962–71. 28880999 10.1111/epi.13895

[42] Slegers R, Wagner L, van Kuijk S, Hilkman D, Hofman P, van Hoof R, et al. Stereo-electroencephalography-guided radiofrequency thermocoagulation restricted to periventricular nodular heterotopias in patients with drug-resistant epilepsy: a single center experience. Seizure. 2024;121:105–13. 39146706 10.1016/j.seizure.2024.07.016

[43] Cossu M, Fuschillo D, Casaceli G, Pelliccia V, Castana L, Mai R, et al. Stereoelectroencephalography-guided radiofrequency thermocoagulation in the epileptogenic zone: a retrospective study on 89 cases. J Neurosurg. 2015;123(6):1358–67. 26090841 10.3171/2014.12.JNS141968

[44] Ryvlin P, Cross JH, Rheims S. Epilepsy surgery in children and adults. Lancet Neurol. 2014;13(11):1114–26. 25316018 10.1016/S1474-4422(14)70156-5

[45] McGovern RA, Ruggieri P, Bulacio J, Najm I, Bingaman WE, Gonzalez-Martinez JA. Risk analysis of hemorrhage in stereo-electroencephalography procedures. Epilepsia. 2019;60(3):571–80. 30746685 10.1111/epi.14668

[46] Vanhatalo S, Voipio J, Kaila K. Full-band EEG (FbEEG): an emerging standard in electroencephalography. Clin Neurophysiol. 2005;116(1):1–8. 15589176 10.1016/j.clinph.2004.09.015

[47] Worrell GA, Jerbi K, Kobayashi K, Lina JM, Zelmann R, Le Van QM. Recording and analysis techniques for high-frequency oscillations. Prog Neurobiol. 2012;98(3):265–78. 22420981 10.1016/j.pneurobio.2012.02.006 PMC3601379

[48] Contento M, Pizzo F, Lopez-Madrona VJ, Lagarde S, Makhalova J, Trebuchon A, et al. Changes in epileptogenicity biomarkers after stereotactic thermocoagulation. Epilepsia. 2021;62(9):2048–59. 34272883 10.1111/epi.16989

[49] Zelmann R, Lina JM, Schulze-Bonhage A, Gotman J, Jacobs J. Scalp EEG is not a blur: It can see high frequency oscillations although their generators are small. Brain Topogr. 2014;27(5):683–704. 24141890 10.1007/s10548-013-0321-y

[50] Frauscher B, von Ellenrieder N, Zelmann R, Rogers C, Nguyen DK, Kahane P, et al. High-frequency oscillations in the normal human brain. Ann Neurol. 2018;84(3):374–85. 30051505 10.1002/ana.25304

[51] Zanello M, Garnier E, Carron R, Jegou A, Lagarde S, Makhalova J, et al. Stereo-EEG-based ictal functional connectivity in patients with periventricular nodular heterotopia-related epilepsy. Epilepsia. 2024;65(4):e47–54. 38345420 10.1111/epi.17891

[52] Pathak A, Ramesh A, Mitra A, Majumdar K. Automatic seizure detection by modified line length and Mahalanobis distance function. Biomed Signal Proces. 2018;44:279–87.

[53] Acharya UR, Fujita H, Sudarshan VK, Bhat S, Koh JEW. Application of entropies for automated diagnosis of epilepsy using EEG signals: a review. Knowl-Based Syst. 2015;88:85–96.

[54] Esteller R, Echauz J, Tcheng T. Comparison of line length feature before and after brain electrical stimulation in epileptic patients. P Ann Int IEEE EMBS. 2004;26:4710–3. 10.1109/IEMBS.2004.1404304 17271360

[55] Usman SM, Khalid S, Akhtar R, Bortolotto Z, Bashir Z, Qiu HY. Using scalp EEG and intracranial EEG signals for predicting epileptic seizures: review of available methodologies. Seizure. 2019;71:258–69. 31479850 10.1016/j.seizure.2019.08.006

[56] Chvojka J, Kudlacek J, Chang WC, Novak O, Tomaska F, Otahal J, et al. The role of interictal discharges in ictogenesis-A dynamical perspective. Epilepsy Behav. 2021;121:106591. 31806490 10.1016/j.yebeh.2019.106591

[57] Shields JA, Greven ACM, Shivamurthy VKN, Dickey AS, Matthews RE, Laxpati NG, et al. Stereoelectroencephalography-guided radiofrequency ablation of the epileptogenic zone as a treatment and predictor of future success of further surgical intervention. Epilepsia. 2023;64(8):2081–93. 37300533 10.1111/epi.17673 PMC11051685

[58] Jehi L, Yardi R, Chagin K, Tassi L, Lo Russo G, Worrell G, et al. Development and validation of nomograms to provide individualised predictions of seizure outcomes after epilepsy surgery: a retrospective analysis. Lancet Neurol. 2015;14(3):283–90. 25638640 10.1016/S1474-4422(14)70325-4

[59] Cortes C, Vapnik V. Support-vector networks. Mach Learn. 1995;20(3):273–97.

[60] Widodo A, Yang BS. Support vector machine in machine condition monitoring and fault diagnosis. Mech Syst Signal Pr. 2007;21(6):2560–74.

[61] Santos MS, Soares JP, Abreu PH, Araújo H, Santos J. Cross-validation for imbalanced datasets: avoiding overoptimistic and overfitting approaches. IEEE Comput Intell M. 2018;13(4):59–76.

[62] Mercier M, Pepi C, Carfi-Pavia G, De Benedictis A, Espagnet MCR, Pirani G, et al. The value of linear and non-linear quantitative EEG analysis in paediatric epilepsy surgery: a machine learning approach. Sci Rep. 2024;14(1):10887. 38740844 10.1038/s41598-024-60622-5 PMC11091060

