# Supplementary material for: A three-dimensional conformal radiofrequency thermocoagulation method for epileptogenic zones in functional cerebral areas: evaluation and prediction of surgical outcomes
Source: Acta Epileptol. 2025 Apr 7;7:25. doi: 10.1186/s42494-025-00214-6 (PMC11974120; doi:10.1186/s42494-025-00214-6)
Supplement: Supplementary file 1 — Supplementary Material 1. [file 42494_2025_214_MOESM1_ESM.docx]

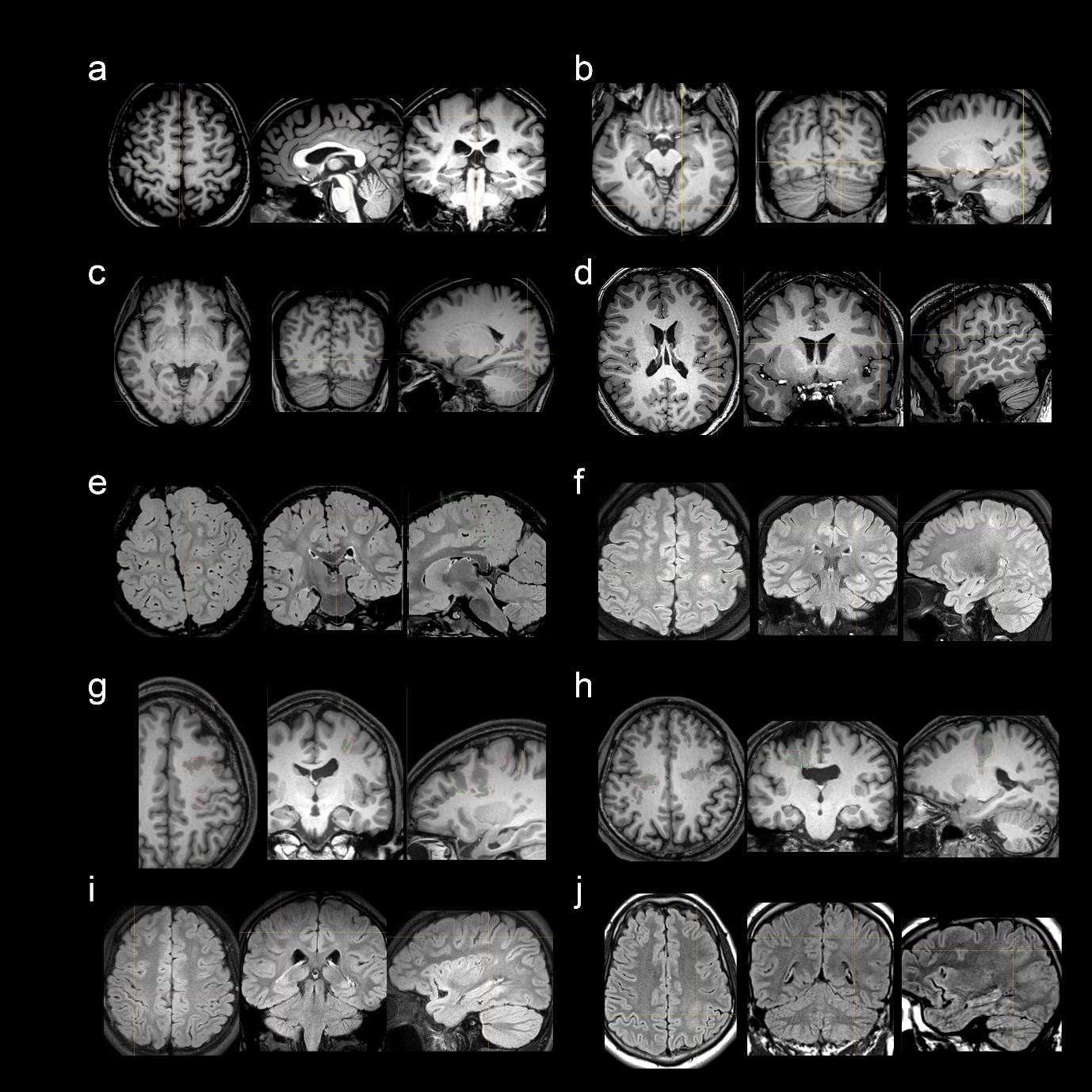


Supplementary Fig. 1. **a**–**j** Preoperative T1-weighted or T2-FLAIR images corresponded to Patient 1–10 in our study respectively. Crossing point of yellow lines in (**a**–**f**, **i**, **j**) focused the location of the structural abnormalities in the images. Red lines in (**e**, **g**, **h**) showed the plan of RF-TC range. Shadows (green or yellow) and green arrows with corresponding letters of contacts in (**e**, **g**, **h**) were location indicators of electrodes and contacts.


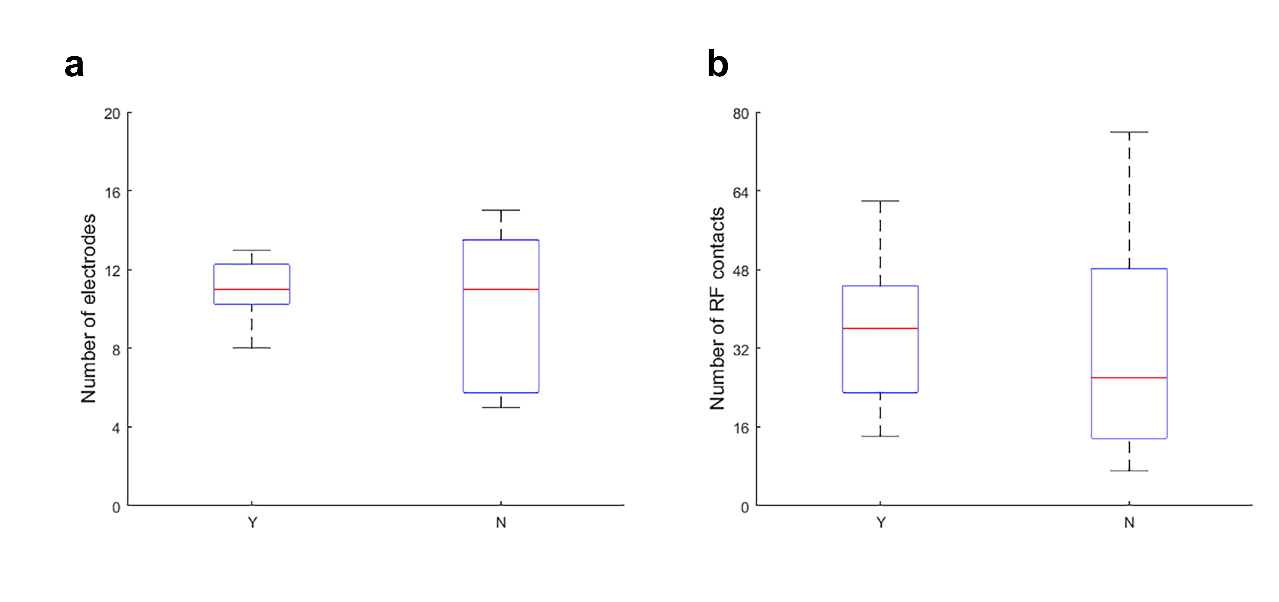


Supplementary Fig. 2. Boxplots of the difference between the number of electrodes (**a**) and RF contacts (**b**) of patients with (*n* = 5) and without short-term functional deficits (*n* = 5). Y, patients with short-term deficits; N, patients without short-term deficits; RF, radiofrequency.
